# CN7:1h Alleviates Inflammation, Apoptosis and Extracellular Matrix Degradation in Osteoarthritis by Modulating the NF‐κB and mTOR Pathways

**DOI:** 10.1111/jcmm.70368

**Published:** 2025-01-28

**Authors:** Chih‐Chien Wang, Jeng‐Wei Lu, Ya‐Wun Wu, You‐Hsiang Chu, Yi‐Jung Ho, Feng‐Cheng Liu, Yi‐Jen Peng

**Affiliations:** ^1^ Department of Orthopedics Tri‐Service General Hospital, National Defense Medical Center Taipei Taiwan; ^2^ Biotech Research and Innovation Centre University of Copenhagen Copenhagen Denmark; ^3^ The Finsen Laboratory, Rigshospitalet/National University Hospital, Faculty of Health and Medical Sciences University of Copenhagen Copenhagen Denmark; ^4^ Graduate Institute of Pathology and Parasitology National Defense Medical Center Taipei Taiwan; ^5^ Graduate Institute of Life Sciences National Defense Medical Center Taipei Taiwan; ^6^ School of Pharmacy, National Defense Medical Center Taipei Taiwan; ^7^ Rheumatology/Immunology and Allergy, Department of Medicine Tri‐Service General Hospital, National Defense Medical Center Taipei Taiwan; ^8^ Department of Pathology Tri‐Service General Hospital, National Defense Medical Center Taipei Taiwan

**Keywords:** chondrocyte, interleukin‐1β, Osteoarthritis, small molecule compound

## Abstract

Osteoarthritis (OA) is a degenerative joint disease with a complex aetiology, which includes inflammation, cellular growth dysregulation and extracellular matrix (ECM) degradation. This study investigated the therapeutic potential of a small‐molecule compound, 2‐amino‐4‐(3,4,5‐trimethoxyphenyl)‐4H‐benzo[h]chromene‐3‐carbonitrile (CN7:1h) in modulating these critical biochemical pathways in OA. Cellular models and rat models of OA were used to explore the impact of CN7:1h on the nuclear factor kappa light chain enhancer of activated B cells (NF‐κB) and mechanistic target of rapamycin (mTOR) signalling pathways. Parameters such as autophagy, apoptosis and ECM preservation were evaluated. CN7:1h demonstrated a non‐cytotoxic profile at a concentration as high as 140 μM as confirmed by 3‐(4,5‐dimethylthiazol‐2‐yl)‐2,5‐diphenyltetrazolium bromide (MTT) assay. At a concentration of 5 μM, CN7:1h was shown to inhibit the activation of NF‐κB and mTOR pathways. CN7:1h was also shown to promote autophagy and reduce apoptosis in cellular models. In rat models, CN7:1h facilitated cartilage repair and demonstrating the therapeutic efficacy of this compound. In conclusion, CN7:1h is a promising bioactive compound for the modulation of key biochemical pathways with therapeutic benefits in degenerative conditions, such as OA. Its high bioavailability and lack of cytotoxicity make CN7:1h an excellent candidate for further research aimed at clinical applications.

## 1 Introduction

Osteoarthritis (OA) is a chronic degenerative joint disorder affecting the elderly worldwide [1, 2]. Characterised by chondrocyte dysfunction, inflammation and progressive degradation of the articular cartilage, OA leads to joint stiffness, pain and the eventual loss of function [3]. The World Health Organisation estimates that 10%–15% of all adults aged over 60 have OA to some degree [4]. It appears that the prevalence increases with age and that the condition is more common in women. Beyond pain and disability, OA imposes a substantial economic burden due to the cost of medical treatment and loss of work productivity.

Most of the therapeutic strategies for OA are palliative (i.e. focusing on pain management and improving joint function) rather than modifying the course of the disease [5]. Conventional treatment options consist primarily of physical therapy, weight management and pharmacologic interventions, which include analgesics (e.g. acetaminophen and non‐steroidal anti‐inflammatory drugs) as well as potent pain relievers (e.g. opioids) for severe cases [6, 7, 8, 9]. Another treatment option is the intra‐articular injection of corticosteroids, hyaluronic acid or platelet‐rich plasma [10, 11]. For patients with advanced disease, surgical intervention (e.g. joint replacement) is often the last resort. Most of these treatments are limited in their capacity to halt or reverse disease progression, and many have potential side effects, particularly after long‐term use.

Considering the limitations of current therapies, there is an urgent need for new treatment strategies to manage OA symptoms and modify the course of the disease. In recent years, there has been growing interest in the development of disease‐modifying OA drugs (DMOADs) [12, 13]. Among the promising candidates in this category are small‐molecule drugs, which can be used to target specific molecular pathways implicated in the pathogenesis of OA [14].

According to previous literature, the nuclear factor kappa light chain enhancer of activated B cells (NF‐κB) signalling is pivotal in mediating inflammatory responses, which significantly contribute to chondrocyte apoptosis, extracellular matrix (ECM) degradation and subsequent cartilage destruction [15]. Mechanistically, NF‐κB functions as a transcription factor, regulating the expression of various pro‐inflammatory cytokines. Given its central role in the inflammatory cascade, NF‐κB signalling has emerged as a promising therapeutic target, with its inhibition demonstrating potential efficacy in mitigating the pathological progression of OA [16]. In addition, current evidence highlights the critical role of the phosphoinositide 3 kinase/AKT/mammalian target of rapamycin (PI3K/AKT/mTOR) signalling pathway in maintaining normal metabolic functions of joint tissues, while also implicating it in the pathogenesis of OA. This pathway regulates key processes such as synovial inflammation, subchondral bone sclerosis, ECM homeostasis and chondrocyte activities, including proliferation, apoptosis, autophagy and inflammatory responses. Dysregulation of these cellular processes accelerates OA progression [17]. Recent studies emphasise the pivotal role of mTOR in cartilage growth, development and the disruption of articular cartilage homeostasis, contributing to cartilage degeneration in OA. Both pharmacological inhibition and genetic deletion of mTOR have been shown to attenuate OA severity in preclinical mouse models, underscoring its therapeutic potential [18].

Small‐molecule drugs typically refer to compounds of low molecular weight, the small size of which allows them to enter cells and interact with intracellular targets, including enzymes, receptors and signalling molecules [19, 20]. Their ease of delivery and ability to be finely tuned for specific targets make them an attractive option for drug development. This study examined a novel small‐molecule drug, 2‐amino‐4‐(3,4,5‐trimethoxyphenyl)‐4H‐benzo[h]chromene‐3‐carbonitrile (CN7:1h), which is a pyridine‐based derivative with potential anti‐catabolic effects in an in vitro interleukin‐1β (IL‐1β)‐induced OA model [21].

This research was based on the hypothesis that CN7:1h modulates key signalling pathways, such as the NF‐κB pathway, which is known to play a critical role in the inflammatory and catabolic responses characteristic of OA. Our objective was to obtain a detailed understanding of the potential therapeutic effects of CN7:1h and its underlying mechanisms. We also sought to lay the groundwork for further development of this compound as a potential DMOAD.

## 2 Materials and Methods

### 2.1 Ethics Statement

This study was approved by the Institutional Review Board (IRB) of Tri‐Service General Hospital, National Defence Medical Center, Taipei, Taiwan (IRB No. 1–102–05‐091, date of approval: 07/25/2016). The research methodology used in this study, which involved human subjects, complied with the Helsinki Declaration of 1964 and any later revisions or equivalent ethical standards, as well as with institutional ethical standards. All experiments involving animals were conducted according to the ethical policies and procedures approved by the Institutional Animal Care and Use Committee (IACUC) of National Defence Medical Center, Taipei, Taiwan (IACUC 17132).

### 2.2 Primary Chondrocyte Culture

Articular cartilage tissue samples were sourced from the knee joints of patients undergoing total knee replacement for OA. All osteoarthritic tissue was carefully removed from joint surfaces, and the remaining cartilage was sectioned into small pieces. These fragments were first incubated in an antimicrobial solution of 500 IU/mL penicillin/streptomycin (Gibco, Carlsbad, CA, USA) for 3 h and then rinsed thoroughly using phosphate‐buffered saline (PBS). Chondrocyte extraction involved a two‐step enzymatic digestion process. The fragments were initially incubated with 0.25% trypsin (Gibco, Carlsbad, CA, USA) under 5% CO_2_ at 37°C for 30 min, followed by 3 mg/mL blend collagenase type H (Sigma‐Aldrich, Merck KGaA, Darmstadt, Germany) under the same conditions for 12 h. The digestion process was monitored under a light microscope. The cell suspension was then collected and filtered through a nylon mesh using a sterile transfer pipette and centrifuged at 1000 rpm/112 rcf (g) for 10 min. The supernatant was discarded, and the resultant cell pellet was resuspended in 10 mL of Dulbecco's Modified Eagle Medium (DMEM)/Nutrient Mixture F‐12 HAM medium (Gibco, Carlsbad, CA, USA) supplemented with 10% foetal bovine serum (FBS) (Gibco, Carlsbad, CA, USA), 100 I.U./mL penicillin (Gibco, Carlsbad, CA, USA), and 100 μg/mL streptomycin (Gibco, Carlsbad, CA, USA). The cultured cells were maintained in a humidified 5% CO_2_ atmosphere at 37°C, using cells from passages 2 to 3 for subsequent experiments.

### 2.3 Cell Viability

Chondrocyte viability was determined via 3‐(4,5‐dimethylthiazol‐2‐yl)‐2,5‐diphenyltetrazolium bromide (MTT) assay (Sigma‐Aldrich, St. Louis, MO, USA). Primary chondrocytes from human OA patients were seeded in 96‐well plates at 1 × 10^4^ cells/well in 200 μL of complete medium. Cells were allowed to adhere overnight prior to treatment with MTT reagent at various concentrations (0, 1, 5, 10, 20, and 40 μM) followed by incubation for 24 or 48 h. Cells treated with 0 μM served as controls, (equating to 100% viability), to which each concentration was compared. Absorbance was measured at 570 nm using a Synergy HT plate reader (Bio‐Tek Instruments Inc., Winooski, VT, USA) [22].

### 2.4 Griess Reaction

Nitric oxide (NO) concentrations in synovial fluid were gauged by measuring the stable end product (nitrite) via the Griess reaction. Briefly, a sample of joint fluid or culture medium was combined with 50 μL of 1% sulphanilamide in 5% phosphoric acid and 50 μL of 0.1% N‐1‐naphthylethylenediamine dihydrochloride. After a 20‐min incubation period at room temperature, absorbance was recorded at 550 nm using a microplate reader (BioTek Instruments, VT, USA) [22].

### 2.5 Protein Extraction and Western Blotting

Cells or tissue samples were quickly rinsed using chilled PBS prior to lysis for 15 min using ice‐cold radioimmunoprecipitation assay (RIPA) buffer (Thermo Fisher Pierce, Waltham, MA, USA) containing 100 μM Na_3_VO_4_ and a 100× protease inhibitor cocktail (Sigma‐Aldrich, St. Louis, MO, USA). Following centrifugation at 13,000/18928 rcf (g) rpm for 15 min, whole cell lysates were harvested to undergo protein concentration assays via the Lowry method. Equivalent quantities of proteins then underwent electrophoresis on a 10% sodium dodecyl sulfate (SDS)‐ polyacrylamide gel before being transferred onto polyvinylidene fluoride (PVDF) membranes (Merck KGaA, Darmstadt, Germany), which were blocked overnight at 4°C using 2% BSA in tris‐buffered saline with Tween 20 (TBST; 12.5 mM Tris/HCl, pH 7.6, 137 mM NaCl and 0.1% Tween 20). Subsequent to three TBST washes, the membranes were probed using primary antibodies specific to the proteins of interest. Specific primary antibodies were purchased from the following commercial suppliers: iNOS (Catalogue number: sc‐651; Santa Cruz Biotechnology, Dallas, TX, USA), MMP‐1 (Catalogue number: ab52631; Abcam, Cambridge, UK), MMP‐2 (Catalogue number: ab97779; Abcam, Cambridge, UK), MMP‐3 (Catalogue number: ab39012; Abcam, Cambridge, UK), MMP‐13 (Catalogue number: ab53015; Abcam, Cambridge, UK), COX‐2 (Catalogue number: RB‐9071; Thermo Fisher Scientific, Waltham, MA, USA), COL2A1 (Catalogue number: ab8887; Abcam, Cambridge, UK), cleaved caspase‐3 (Catalogue number: #9661; Cell Signalling Technology, Danvers, MA, USA), mTOR (Catalogue number: #2972; Cell Signalling Technology, Danvers, MA, USA), p‐mTOR (Catalogue number: #2971; Cell Signalling Technology, Danvers, MA, USA), LC3 I/II (Catalogue number: #4108; Cell Signalling Technology, Danvers, MA, USA), β‐actin (Catalogue number: sc‐47,778; Santa Cruz Biotechnology, Dallas, TX, USA) and GAPDH (Catalogue number: 5174S; Cell Signalling Technology, Danvers, MA, USA). After three more TBST washes, the membranes were incubated with horseradish peroxidase (HRP)‐conjugated secondary antibodies at room temperature for 1h. Extensive rewashing was performed prior to detection using enhanced chemiluminescence Western blotting detection reagents (Merck Millipore, WBLUR0500, Darmstadt, Germany), as per the manufacturer's guidelines. Finally, the membranes were scanned, and the results were quantified via densitometry [23].

### 2.6 Enzyme‐Linked Immunosorbent Assay (ELISA)

An ELISA kit (R&D Systems, Minneapolis, MN, USA) was used to quantify cytokine expression levels in tissue fluids or culture media in accordance with the guidelines outlined by the manufacturer [22].

### 2.7 Gelatin Zymography

Extracts were combined with a sample buffer that included SDS, glycerol and bromophenol blue. Equal quantities of each sample were separated on an 8% SDS‐polyacrylamide gel containing 1 mg/mL of gelatin. Following SDS‐polyacrylamide gel electrophoresis (SDS‐PAGE), the gels were washed twice using 2.5% Triton X‐100 for 30 min to remove the SDS. They were then washed twice more using distilled water and equilibrated using incubation buffer consisting of 100 mM Tris/HCl, 30 mM CaCl_2_ and 0.01% NaN_3_. The gel was subsequently incubated in incubation buffer for 20 h at 37°C prior to staining with Coomassie Blue solution for 40 min. De‐staining was performed using a solution of methanol, acetic acid and distilled water [24].

### 2.8 Safranin O—Fast Green Staining

Slides were baked at 75°C for 30 min for deparaffinisation and then hydrated via a series of ethanol solutions at concentrations of 70%, 80%, 95% and 100%. The slides were then stained using Weigert's iron haematoxylin working solution (Catalogue number:H3136; Sigma‐Aldrich, St. Louis, MO, USA) at room temperature for 5 min. They were then gently washed under running tap water for 5 min before being stained with a 0.02% Fast Green solution (Catalogue number: F7258; Sigma‐Aldrich, St. Louis, MO, USA) for another 5 min. The slides were subsequently briefly rinsed in 1% acetic acid for 10 s before being transferred to a 1% Safranin O solution (Catalogue number: 2255; Sigma‐Aldrich, St. Louis, MO, USA) for 3 min. Finally, the slides were carefully rinsed in two changes of 95% alcohol and air‐dried [24].

### 2.9 Electrophoretic Mobility Shift Assay

An electrophoretic mobility shift assay (EMSA) for the detection of DNA binding activity of NF‐κB was performed using a commercial kit (LightShift Chemiluminescent EMSA kit, Thermo, USA). Biotin end‐labelled duplex DNA was incubated with nuclear extract and then electrophoresed on a native gel. The reaction mixtures were separated on a 5% native polyacrylamide gel at 4°C for 1h and subsequently transferred to a nylon membrane (GE Healthcare, Buckinghamshire, UK). The biotin end‐labelled DNA was probed with a streptavidin‐HRP conjugate followed by the application of ECL reagents. Finally, the membranes were exposed to UV light for 10 min, and the results were analysed using a UVP system [24].

### 2.10 Assessment of Cellular Apoptosis via Flow Cytometry

Cellular apoptosis was examined via flow cytometry using an apoptosis assay with FITC Annexin V (BD Pharmingen, San Jose, CA, USA) and propidium iodide (PI). Cells were centrifuged at 400 g for 5 min and divided into four groups: Control, CN7:1h, IL‐1β, and CN7:1h + IL‐1β. Each sample was subsequently resuspended in 100 μL of 1× binding buffer and stained with 5 μL of FITC Annexin V and 5 μL of PI. The samples were then gently vortexed and incubated at room temperature in the dark for 15 min. To each sample, 400 μL of 1× binding buffer was added to undergo flow cytometry analysis within 1h. This method enabled the differentiation of early and late apoptotic cells as well as live cells [25].

### 2.11 Ex Vivo Cartilage Treatment and Processing

Residual cartilage specimens from total knee arthroplasty were washed using sterile PBS for the removal of bone fragments and blood residue. The tissues were classified into Control, CN7:1h, IL‐1β and CN7:1h + IL‐1β groups and then incubated it, ex vivo for 48 h. Following incubation, the tissue samples were fixed in formalin for 4–6 h, dehydrated using a tissue processor, and embedded in paraffin. Using a sliding microtome, initial 10 μm‐sections were removed until the cartilage surface was fully exposed, after which 3 μm‐sections were sampled and floated in cold water prior to placement on blank slides submerged in 55°C hot water to fix the tissue. To promote adhesion, the slides were then baked at 75°C for 15–30 min [22].

### 2.12 Animal Experiment

Male Sprague Dawley rats were sourced from BioLASCO Taiwan Co. Ltd. (Yi‐Lan, Taiwan) and acclimatised for 7 days. All experimental procedures were approved by the IACUC at the National Defence Medical Center under Protocol No. IACUC‐17‐132. At 8 weeks old, the rats were randomly allocated into three groups: control, injury and treatment. Rats in the injury and treatment groups underwent anterior cruciate ligament transection (ACLT) to simulate joint instability. Surgical incisions were closed using sutures post‐operation. Rats in the treatment group received oral gavage of CN7:1h at a dose of 40 mg/kg, administered three times per week. This design made it possible to study the effects of CN7:1h on knee joint injuries induced via ACLT. Note that the rats oral gavage method was based on previous research [26].

### 2.13 Haematoxylin and Eosin (H&E) Staining

Sections prepared from tissue blocks were stained using haematoxylin for 5 min and eosin for 3 min at room temperature for histological examination. The slides that underwent H&E staining were observed under a light microscope at magnifications of 100× and 200×. The Osteoarthritis Research Society International (OARSI) scoring system was used to grade the severity and extent of OA in the articular cartilage with total scores ranging from 0 to 6 [27].

### 2.14 Statistical Analysis

All data were averaged from at least three independent experiments. Data are represented as the mean ± standard deviation (SD). The ANOVA test with Dunnett post hoc test was chosen for comparing more than two groups allowing for the nonsymmetrical distribution data. A *p*‐value of less than 0.05 indicated statistical significance [28].

## 3 Results

### 3.1 Viability of Chondrocytes Under CN7:1h Treatment

To determine a safe concentration range of CN7:1h for chondrocytes, an initial MTT assay was conducted to establish concentrations at which the CN7:1h exhibited cytotoxicity (Figure S1). Primary chondrocyte cells were seeded in 96‐well plates at a density of 1 × 10^4^ cells/well. CN7:1h concentrations at 0, 1, 5, 10, 20 and 40 μM were selected for further testing over periods of 24 and 48 h. There were no statistically significant differences in cell viability between the control and any of the concentrations, irrespective of the duration of incubation.

### 3.2 Effect of CN7:1h on Inflammatory Markers, MMP Expression and NO Release in Chondrocytes Following IL‐1β Stimulation

**FIGURE 1:**
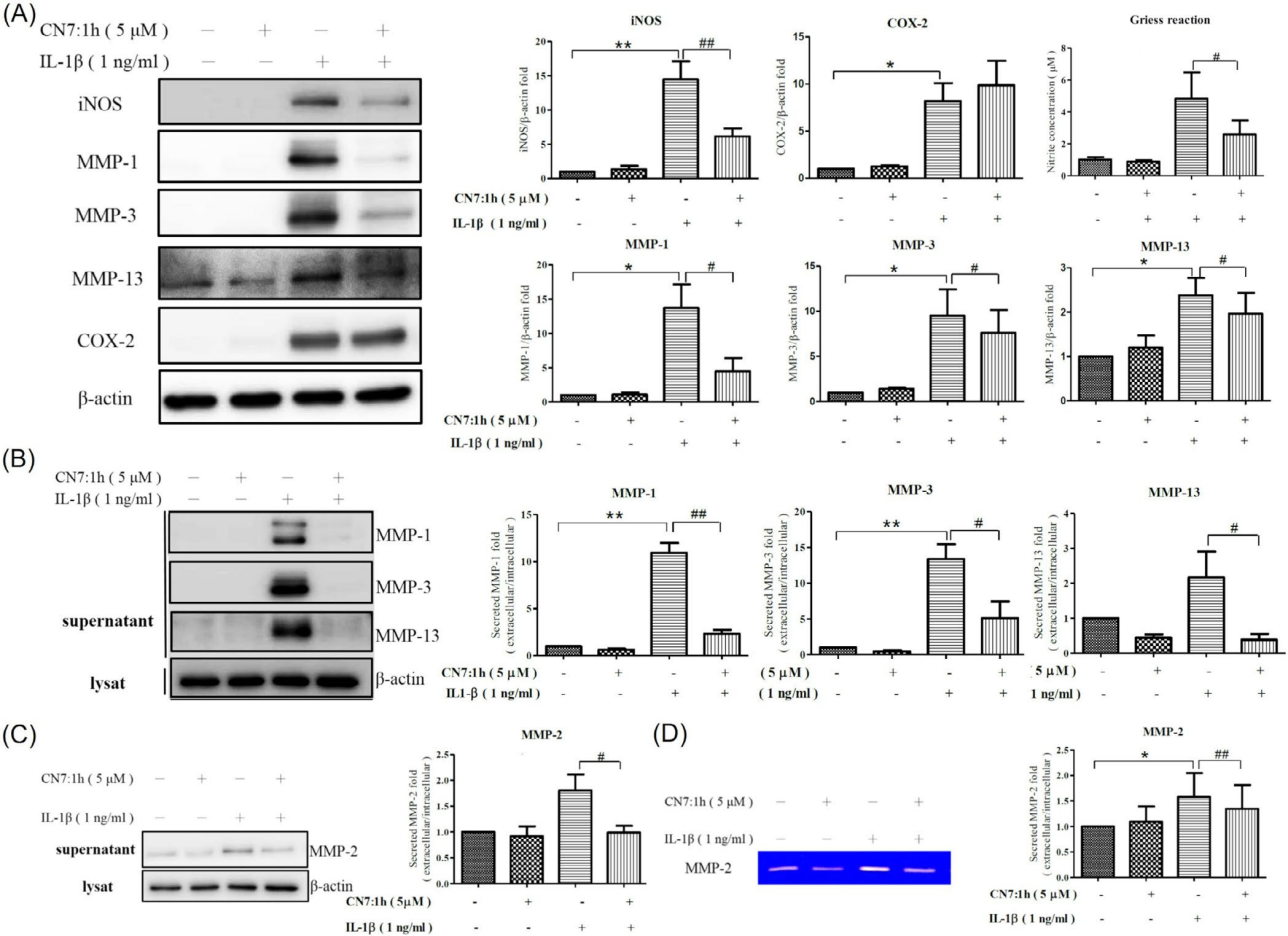
Effects of CN7:1h pretreatment on IL‐1β‐induced protein expression and nitric oxide production in chondrocytes. (A) Chondrocytes were pretreated with 5 μM CN7:1h for 2 h and then co‐cultured with IL‐1β (1 ng/mL) for 24 h. Western blotting was conducted to analyse the expression levels of iNOS, COX‐2, MMP‐1, MMP‐3 and MMP‐13 in cell lysate, with β‐Actin serving as an internal control. Semi‐quantitative data are presented in bar graphs (*p < 0.05, **p < 0.01 compared to control; ^*#*^ *p* < 0.05 compared to IL‐1β group). The supernatant was collected to measure NO concentrations using the Griess reaction (*p < 0.05 compared to control; ^*#*^ *p* < 0.05 compared to IL‐1β group). (B) After 2‐h pretreatment with 5 μM CN7:1h and 24‐h co‐culturing with IL‐1β (1 ng/mL), supernatants were collected for Western blot analysis of MMP‐1, MMP‐3 and MMP‐13 (**p < 0.01 compared to control; ^*#*^ *p* < 0.05, ^*##*^ *p* < 0.01 compared to IL‐1β group). (C) Western blot analysis of secreted MMP‐2 expression produced semi‐quantitative data, presented in the form of bar graphs (*p < 0.05 compared to control; ^*#*^ *p* < 0.05, ^##^ *p* < 0.01 compared to IL‐1β group). (D) Gelatin zymography produced semi‐quantitative data, presented in the form of bar graphs (*p < 0.05 compared to control; ^#^ *p* < 0.05, ^##^ *p* < 0.01 compared to IL‐1β group). Significant differences were detected used ANOVA test with Dunnett post hoc test.

Chondrocytes were pretreated with 5 μM CN7:1h for 2 h and then co‐incubated with IL‐1β (1 ng/mL) for 24 h. The protein expression levels of iNOS, COX‐2, MMP‐1, MMP‐3 and MMP‐13 in the cell lysate were evaluated using Western blot analysis (Figure 1A). Compared to the control, IL‐1β stimulation resulted in a significant increase in the protein expression levels of NOS (**p < 0.01), COX‐2 (*p < 0.05), MMP‐1 (*p < 0.05), MMP‐3 (*p < 0.05) and MMP‐13 (*p < 0.05) in the chondrocytes. Moreover, the group pretreated with 5 μM CN7:1h for 2 h exhibited a noticeable reduction in iNOS expression levels (^##^ *p* < 0.01). No significant changes were observed in COX‐2 levels under the effects of 5 μM CN7:1h. Using the pretreatment and co‐incubation procedure for IL‐1β, we collected cell culture media to assess the effect of CN7:1h on NO release via the Griess reaction.

Compared to the control group, IL‐1β stimulation resulted in a increase in NO levels. Relative to the IL‐1β group, the group pretreated with 5 μM CN7:1h for 2 h exhibited a marked decrease in NO levels (^#^ *p* < 0.05). Further analysis revealed elevated expression levels of MMP1, 3, and 13 in the cell culture medium under IL‐1β stimulation. Note that the medium from the group pretreated with 5 μM CN7:1h for 2 h presented a significant reduction in the expression levels of MMP‐1 (^#^ *p* < 0.05), MMP‐3 (^##^ *p* < 0.01) and MMP‐13 (^#^ *p* < 0.05) (Figure 1B).

### 3.3 Assessment of CN7:1h Influence on MMP 2 Secretion in Human Chondrocytes

Chondrocytes were pretreated with 5 μM CN7:1h for 2 h, followed by co‐incubation with IL‐1β (1 ng/mL) for 24 h. The cell culture supernatant was then collected to assess the effects of CN7:1h on chondrocyte‐secreted MMP‐2 using Western blot analysis and gelatin zymography (Figure 1C,D). At baseline (without stimulation), chondrocytes secreted a basal quantity of MMP 2. Stimulation with IL‐1β significantly increased the secretion of MMP‐2 by chondrocytes, exceeding the control group (*p < 0.05). MMP 2 secretion by chondrocytes in the group pretreated with 5 μM CN7:1h for 2 h was lower than that in the IL‐1β group (^#^ *p* < 0.05) (^##^ *p* < 0.01).

### 3.4 COL2A1 Expression Influenced by CN7:1h in Human Chondrocytes

**FIGURE 2:**
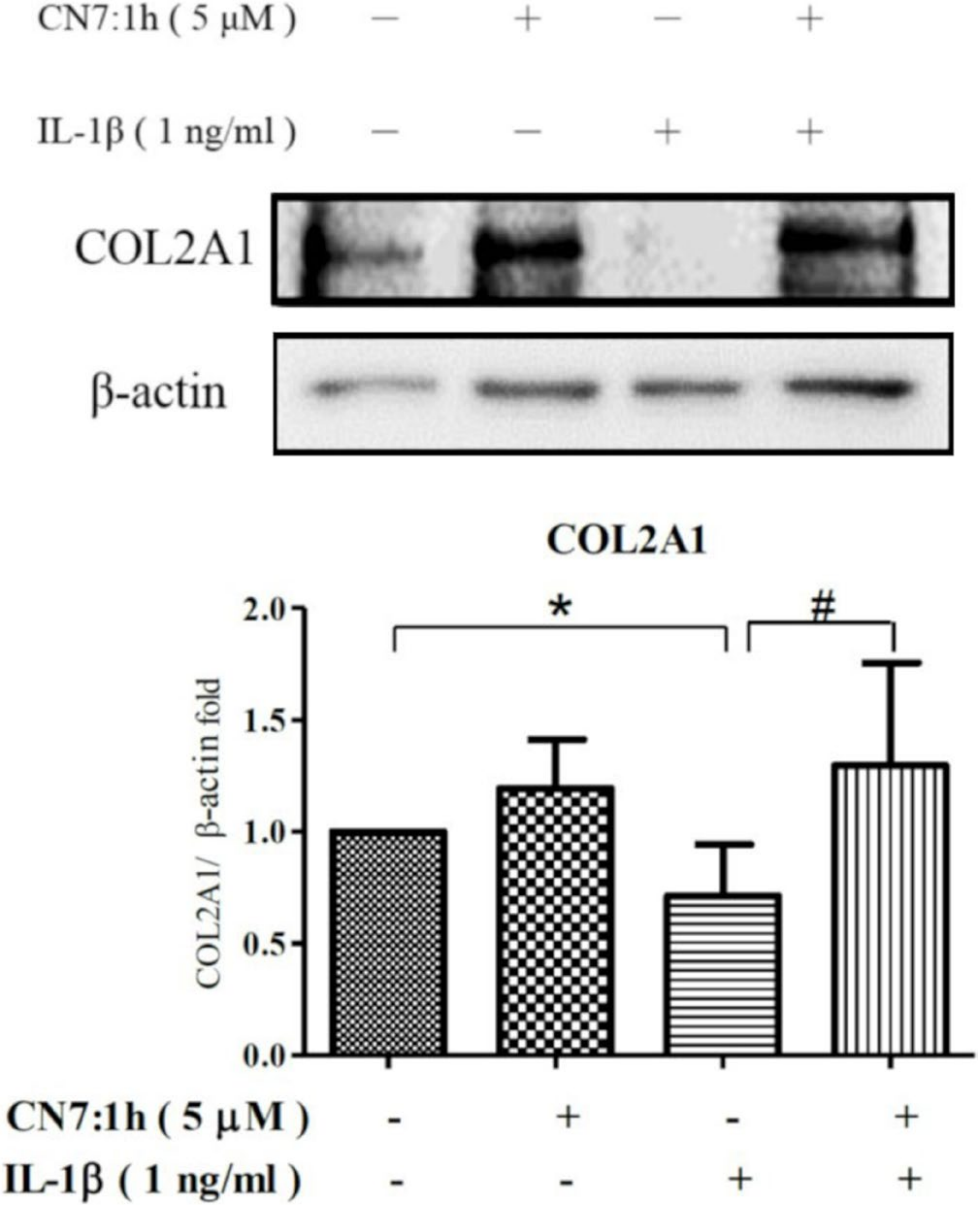
Influence of CN7:1h on collagen II protein expression in chondrocytes subjected to IL‐1β stimulation. Chondrocytes were pretreated with 5 μM CN7:1h for 2 h and then co‐cultured with IL‐1β (1 ng/mL) for an additional 24 h. Western blot analysis was used to analyse the protein expression levels of collagen II in cell lysate. Semi‐quantitative data are represented in the form of bar graphs, presented as mean ± SD (*p < 0.05 compared to the control group; ^#^ *p* < 0.05 compared to the IL‐1β group). Significant differences were detected used ANOVA test with Dunnett post hoc test.

Chondrocytes were pretreated with 5 μM CN7:1h for 2 h and subsequently co‐incubated with IL‐1β (1 ng/mL) for 24 h. Whole‐protein extracts were then obtained for the evaluation of COL2A1 protein expression using Western blot analysis (Figure 2). Under stimulation with only IL‐1β, the expression of COL2A1 protein was significantly lower (*p < 0.05) than that of the control group. COL2A1 expression in the group pretreated with 5 μM CN7:1h for 2 h was significantly higher (^#^ *p* < 0.05) than in the IL‐1β group.

### 3.5 Investigating CN7:1h‐Induced Apoptotic Changes in Human Chondrocytes

**FIGURE 3:**
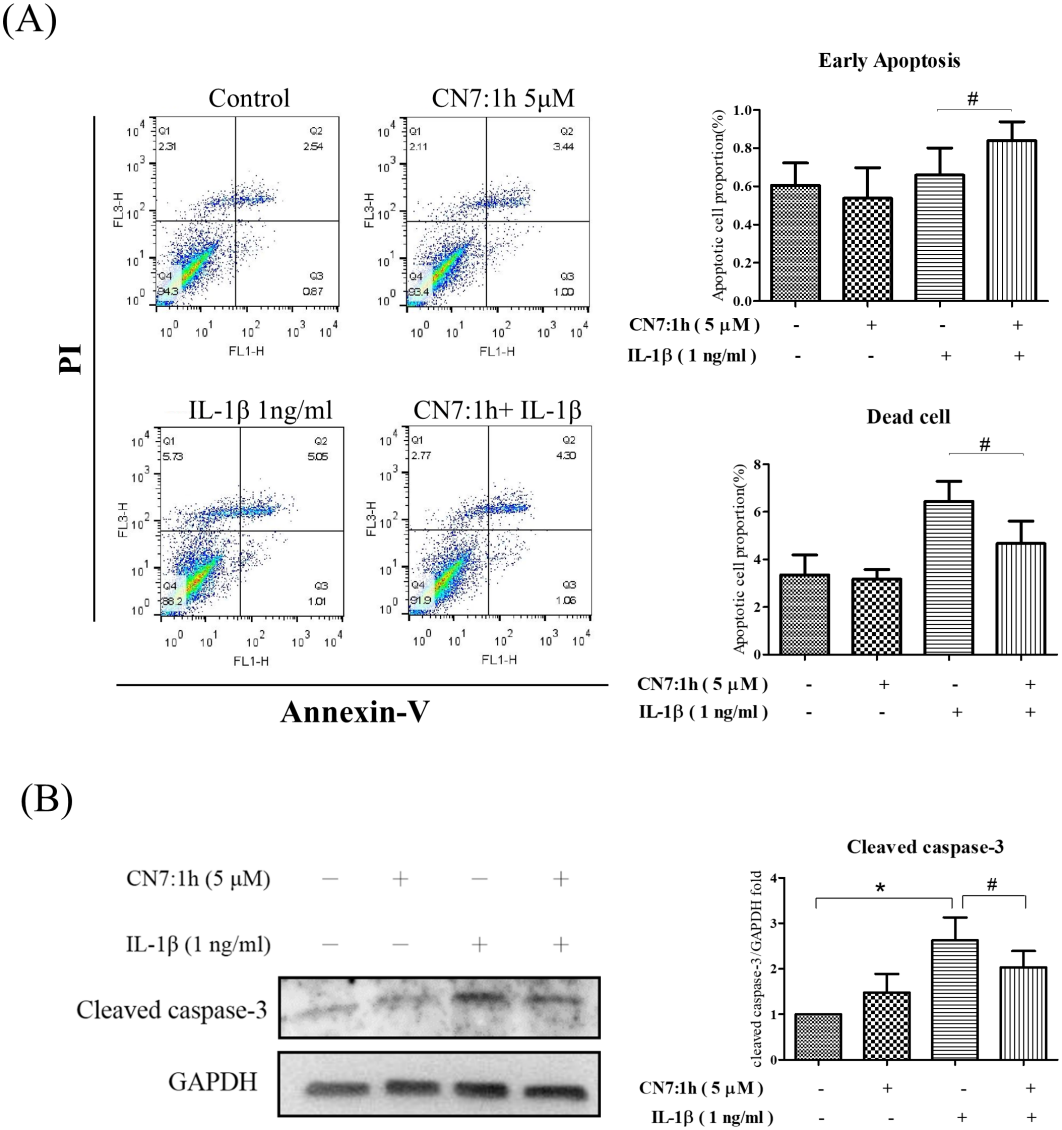
Effects of CN7:1h on apoptotic pathways in human chondrocytes subjected to IL‐1β stimulation. (A) Early apoptosis levels were significantly higher in chondrocytes pretreated with 5 μM CN7:1h for 2 h than in the IL‐1β‐treated group (^#^ *p* < 0.05), which was accompanied by a slowed progression toward late apoptosis. Pretreatment with 5 μM CN7:1h also resulted in a notable decrease in the number of dead cells (^#^ *p* < 0.05). (B) Cleaved caspase‐3 levels in cells subjected to IL‐1β stimulation alone were significantly higher than in the control group (*p < 0.05). Pretreatment with 5 μM CN7:1h for 2 h followed by co‐culturing with IL‐1β (1 ng/mL) significantly decreased the expression of cleaved caspase‐3 (^#^ *p* < 0.05). Significant differences were detected used ANOVA test with Dunnett post hoc test.

In this experiment, chondrocytes were maintained in a serum‐free environment for 2 h and were then pretreated with 5 μM CN7:1h for an additional 2 h. After adding IL‐1β (1 ng/mL), the cells were co‐incubated for 24 h. Cell apoptosis was then assessed using flow cytometry after staining with Annexin V FITC and PI (Figure 3A). Early apoptosis in the group pretreated with 5 μM CN7:1h for 2 h was significantly higher (^#^ *p* < 0.05) than in the IL‐1β group. This indicated a slowed progression toward late apoptosis. Furthermore, the number of dead cells was significantly reduced (^#^ *p* < 0.05). After co‐incubating the cells with IL‐1β (1 ng/mL) and 5 μM CN7:1h for 24 h, whole‐protein extracts were obtained to determine the expression of cleaved caspase‐3 using Western blot analysis (Figure 3B). The expression of cleaved caspase‐3 in cells stimulated solely with IL‐1β was significantly higher (*p < 0.05) than in the control group. Pretreating cells with 5 μM CN7:1h for 2 h significantly decreased (^#^ *p* < 0.05) cleaved caspase‐3 expression levels.

### 3.6 Effects of CN7:1h on p‐mTOR Protein Levels and Autophagy in Human Chondrocytes

**FIGURE 4:**
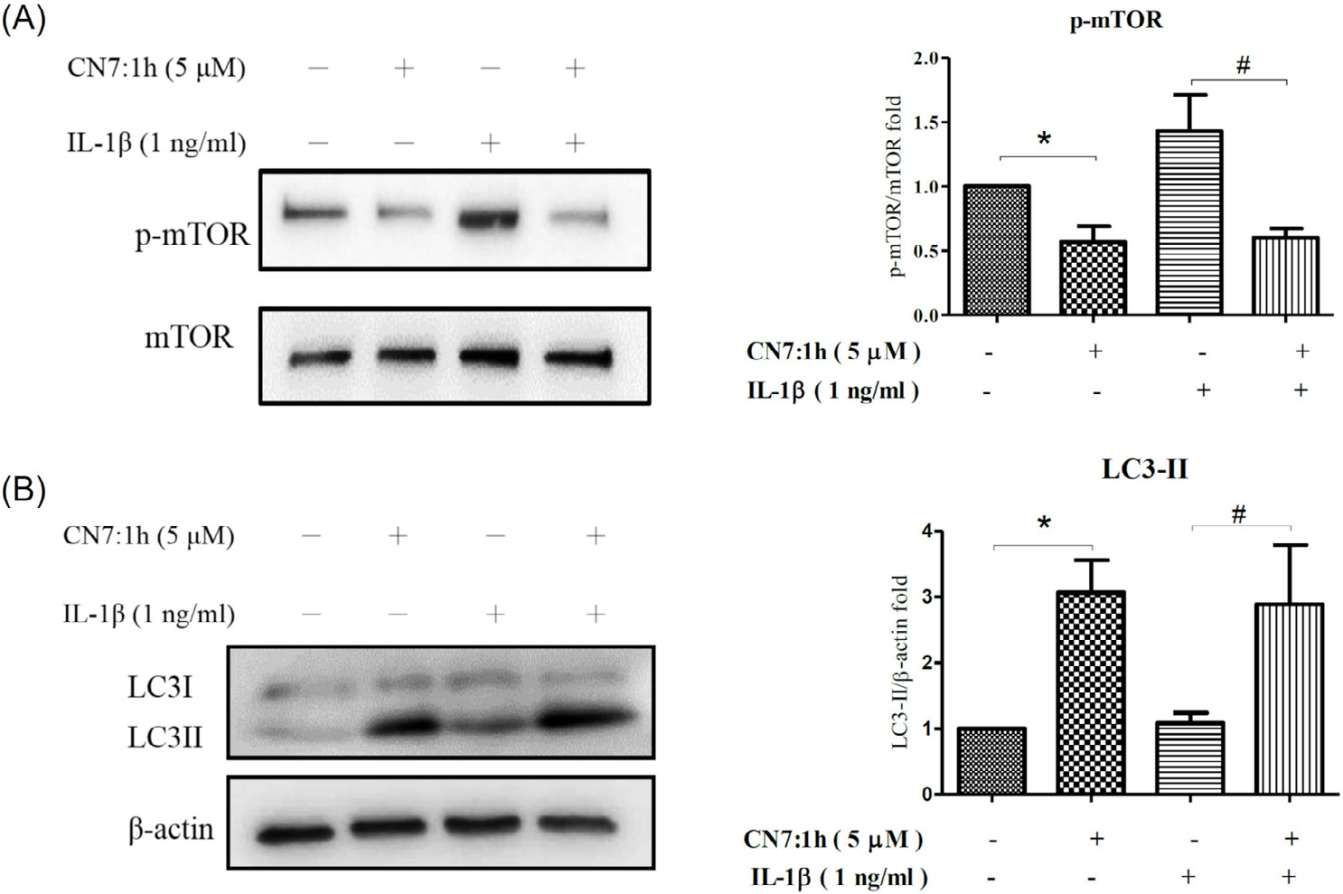
Effects of CN7:1h on p‐mTOR and autophagy in human chondrocytes exposed to IL‐1β. Chondrocytes were pretreated with 5 μM CN7:1h for 2 h and then co‐cultured with IL‐1β (1 ng/mL) for 24 h. Protein expression was analysed using Western blot analysis. (A) p‐mTOR levels in the presence of only CN7:1h were significantly lower than in the control group (*p < 0.05). Co‐culturing the cells with IL‐1β after pretreatment with 5 μM CN7:1h significantly reduced p‐mTOR levels (^#^ *p* < 0.05). (B) LC3 II expression in cells exposed only to CN7:1h was significantly higher than in the control group (*p < 0.05). Co‐culturing the cells with IL‐1β after pretreatment with 5 μM CN7:1h significantly increased LC3 II expression (^#^ *p* < 0.05). Significant differences were detected used ANOVA test with Dunnett post hoc test.

Chondrocytes were pretreated with 5 μM CN7:1h for 2 h and then co‐incubated with IL‐1β (1 ng/mL) for 24 h. Whole‐protein extracts were then isolated to determine the expression levels of phosphorylated mTOR (p‐mTOR) and autophagy markers LC3 I and LC3 II using Western blot analysis (Figure 4). p‐mTOR levels expression levels in cells treated only with CN7:1h (*p < 0.05) or pretreated with 5 μM CN7:1h (^#^ *p* < 0.05) for 2 h was significantly decreased than in the control group or IL‐1β group (Figure 4A). LC3 II levels in cells treated only with CN7:1h were significantly upregulated, exceeding those in the control group (*p < 0.05). LC3 II levels in the group pretreated with 5 μM CN7:1h for 2 h were significantly upregulated, exceeding those in the IL‐1β treated group (^#^ *p* < 0.05) (Figure 4B).

### 3.7 Effects of CN7:1h on Transcription Factor Activation and Inhibition in Chondrocytes

**FIGURE 5:**
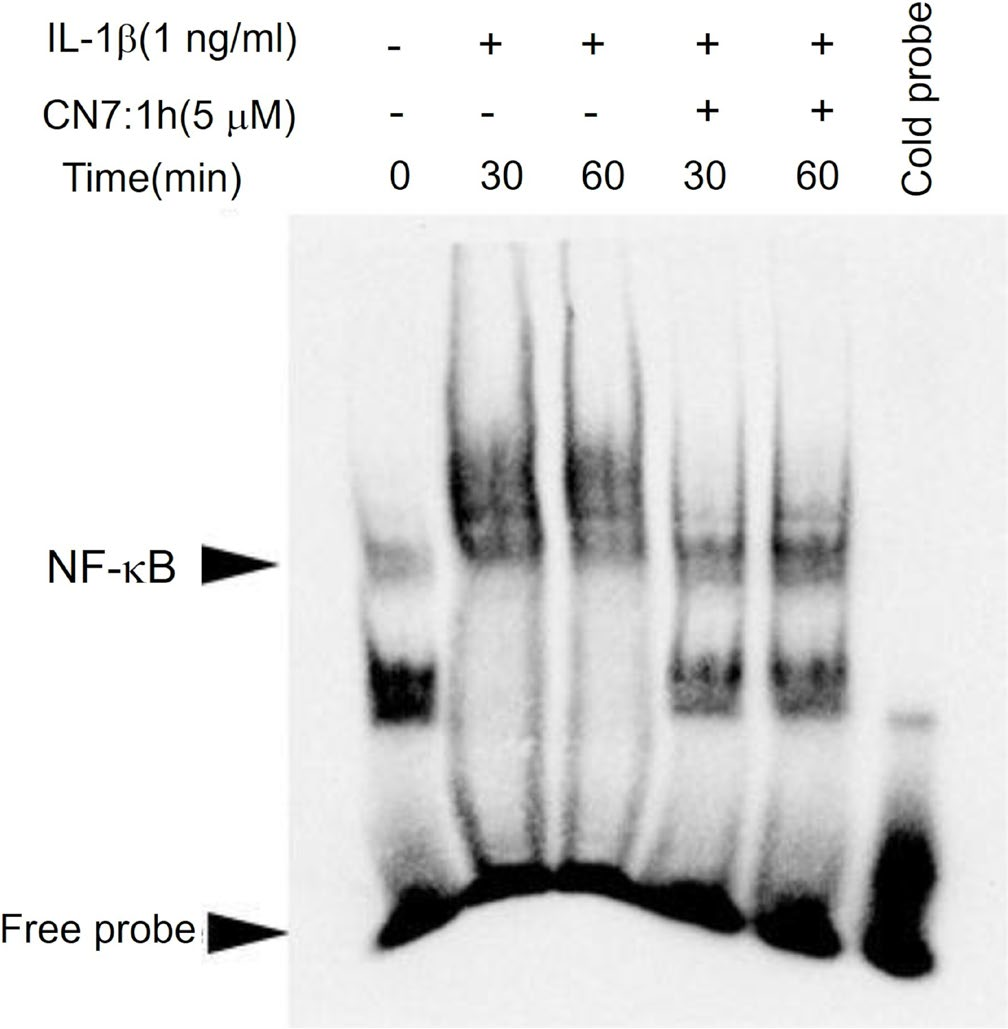
Impact of CN7:1h on transcription factors. Chondrocytes were pretreated with 5 μM of CN7:1h for 2 h and then stimulated using IL‐1β (1 ng/mL) for 30 and 60 min. Nuclear proteins were extracted from the chondrocytes and analysed using EMSA to determine the expression levels of the transcription factor NF‐κB translocating into the nucleus. In the untreated group, we observed an increase in activated NF‐κB binding to DNA after IL‐1β stimulation for 30 min. The quantity of NF‐κB translocating into the nucleus in the groups pretreated with 5 μM of CN7:1h for 2 h was less than in the untreated groups, at 30 and 60 min, thereby indicating that CN7:1h is capable of inhibiting activation of the transcription factor NF‐κB.

Chondrocytes with or without 2‐h pretreatment using 5 μM CN7:1h underwent stimulation with 1 ng/mL IL‐1β for 30 or 60 min. Nuclear protein extracts from chondrocytes were subjected to EMSA to examine the nuclear translocation of the transcription factor NF‐κB (Figure 5). In the untreated group, 30‐min IL‐1β stimulation resulted in the activation of NF‐κB, as indicated by an increase in the quantity of NF‐κB binding to DNA. In the CN7:1h pretreatment group, the nuclear translocation of NF‐κB was noticeably lower at both time points (30 and 60 min). These results indicate that CN7:1h has an inhibitory effect on the activation of NF‐κB.

### 3.8 CN7:1h‐Induced Alterations in the Extracellular Matrix in Ex Vivo Cartilage Model

**FIGURE 6:**
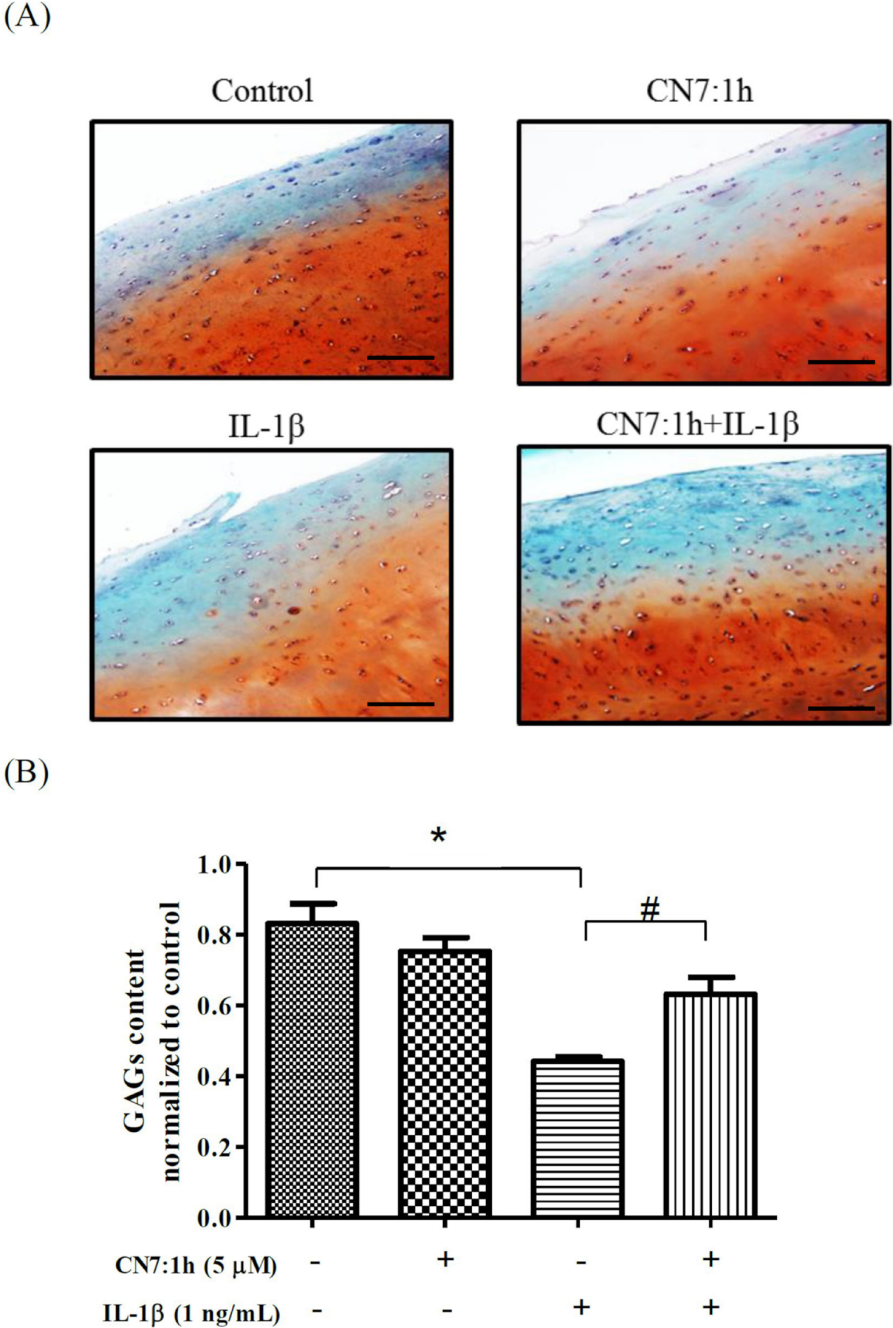
Effects of CN7:1h on GAG content in cartilage tissue. Following the ex vivo cultivation of cartilage tissue, Alcian Blue‐Safranin O staining was used to assess the loss of the ECM. (A) Images captured at 100× magnification were used to examine the integrity of the ECM. (B) Semi‐quantitative bar graphs indicate GAG content in the cartilage tissue. GAG content was significantly higher in the control group (*p < 0.05) and the CN7:1h with IL‐1β‐treated group (^#^ *p* < 0.05) than in the IL‐1β‐treated group, respectively. Scale bars = 50 μm. Significant differences were detected used ANOVA test with Dunnett post hoc test.

We sought to elucidate the effects of CN7:1h on the ECM in an ex vivo cartilage model. We observed no statistically significant difference between the CN7:1h group and the control group in terms of glycosaminoglycans (GAGs) content. The IL‐1β group presented a significant decrease in GAG (*p < 0.05). Note that pretreatment with 5 μM CN7:1h for 2 h appears to have moderated IL‐1β‐induced GAG loss (^#^ *p* < 0.05) (Figure 6A,B).

### 3.9 Impact of CN7:1h on Rat Knee Joint Repair

**FIGURE 7:**
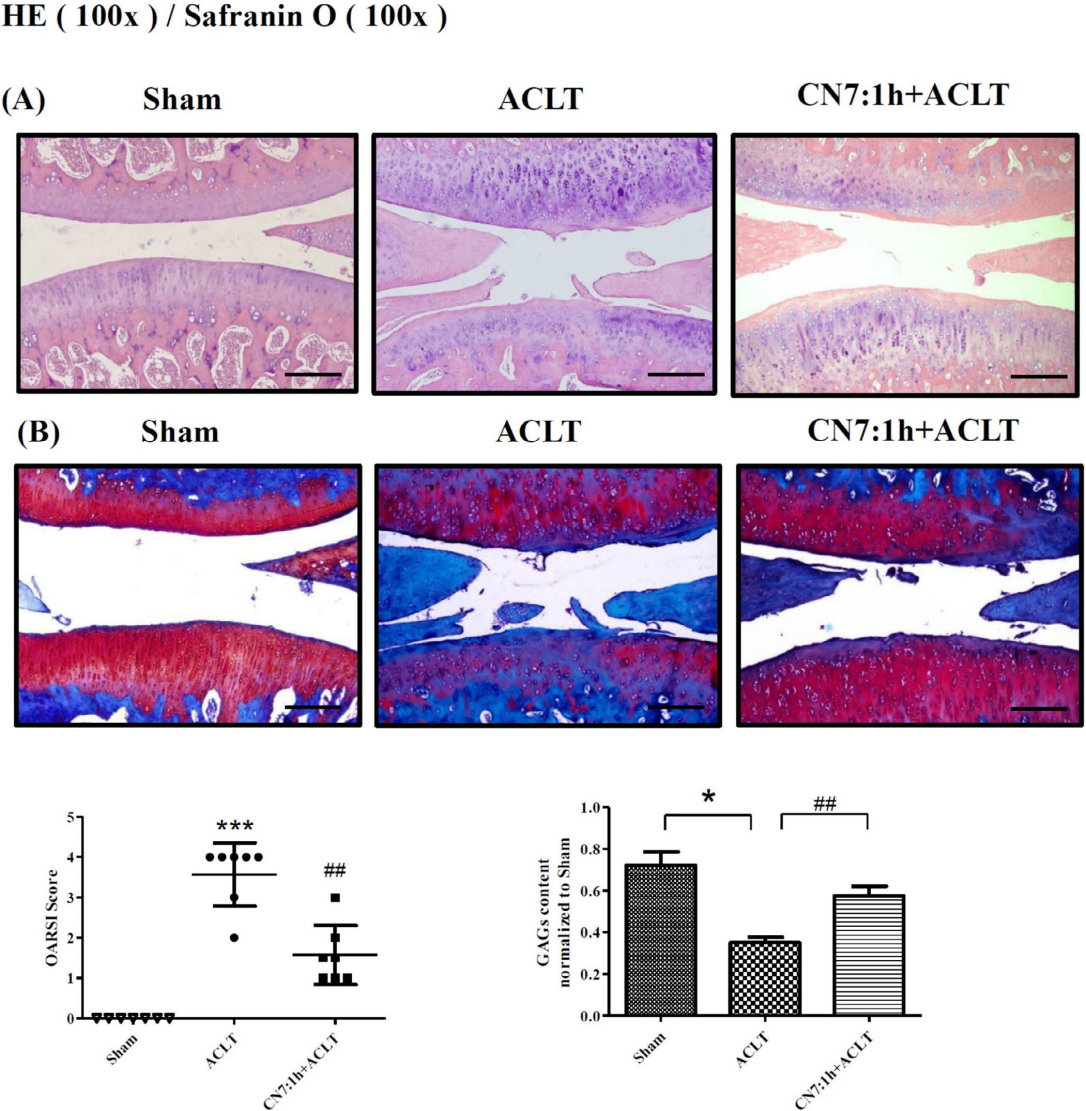
Effects of CN7:1h treatment on cartilage repair and tissue changes in a rat model. (A) Images of H&E‐stained samples captured under 100× magnification revealed significant cartilage damage in the ACLT injury group, far exceeding that in the Sham group (***p < 0.001). Treatment with CN7:1h was shown to preserve cartilage compared to the ACLT injury group (^##^ *p* < 0.01). (B) Images of Alcian Blue‐Safranin O samples captured at 100× magnification were used to evaluate the loss of GAGs. GAG levels were significantly lower in the ACLT injury group than in the Sham group (*p < 0.05). Treatment with CN7:1h significantly reduced GAG loss compared to the ACLT injury group (^##^ *p* < 0.01). Scale bars = 50 μm. Significant differences were detected used ANOVA test with Dunnett post hoc test.

H&E staining revealed that wear on the surface of cartilage was far more severe in the injury group than in the sham group. CN7:1h‐treated ACLT rats presented a return to smooth, normal cartilage surfaces (Figure 7A). OARSI scores for CN7:1h‐treated ACLT rats were significantly higher than in the injury group (^##^ *p* < 0.01). Safranin O staining and Image J analysis at magnifications of 100× and 200× confirmed that GAG levels in the CN7:1h group were significantly higher than in the injury group (^##^ *p* < 0.01) (Figure 7B).

## 4 Discussion

CN7:1h exhibited inhibitory effects on the NF‐κB and mTOR signalling pathways, which play pivotal roles in regulating inflammation and cellular growth. These modulatory effects led to an increase in autophagy levels and a decline in apoptosis, both of which could have a profound effect on cellular repair and tissue regeneration, particularly in degenerative diseases, such as OA. Degenerative OA is a chronic joint disorder characterised by progressive cartilage degradation, subchondral bone remodelling and synovial inflammation [5, 29]. A hallmark of OA pathogenesis is an increase in the production of inflammatory mediators (e.g. IL‐1β), tumour necrosis factor α and various matrix metalloproteinases (MMPs) [3, 30]. These biomolecules complicate the management of this disease by promoting cartilage erosion, impairing joint function and increasing pain.

Most conventional treatment options for OA focus on pain management and improving joint mobility, often through the use of nonsteroidal anti‐inflammatory drugs or corticosteroid injections [6, 7, 9, 31]. Note, however, that those interventions do not address the underlying pathology and may even exacerbate cartilage breakdown over time. In the current study, we focused on a novel small molecule synthesised by Lee et al. [21]. Many small‐molecule drug compounds modulate key biochemical pathways. They are generally easy to administer (e.g. orally) and provide high bioavailability, as evidenced by their rapid diffusion into cells. Nonetheless, small molecules are also strongly associated with off‐target effects requiring rigorous investigation. In assessing the therapeutic potential and safety of any new small molecule, such as CN7:1h, it is essential to determine the specificity and mechanisms underlying the observed effects. Our initial in vitro analysis using MTT assays revealed no cytotoxic effects at a concentration of 1–40 μM. The absence of cytotoxicity is a crucial indicator of the potential therapeutic applicability of this compound. Note that low concentrations (e.g. 1 μM) were ineffective; therefore, we selected 5 μM for all subsequent experiments (Figure S1).

OA remains a complex degenerative condition affected by age, joint injury and obesity [5, 31]. The key role of inflammation is mediated primarily by chondrocytes and synovial cells [3, 30], which promote disease progression by releasing cytokines and chemokines into the synovial fluid. Pretreatment with CN7:1h significantly mitigated IL‐1β‐induced downregulation of COL2A1 and inhibited the activation of key transcription factors such as NF‐κB. This downregulated the expression of inflammation‐related proteins and decreased the levels of oxidative stress markers (Figure 2). Autophagy is a metabolic pathway involved in maintaining joint homeostasis. Previous researchers have suggested that the age‐related decrease in autophagy levels contributes to age‐related diseases, such as OA [3, 32]. Pretreatment with CN7:1h led to a notable decrease in p‐mTOR levels, which implies an increase in autophagy. This opens a new therapeutic avenue for the treatment of OA (Figure 4). Our experiments also revealed that pretreatment with CN7:1h significantly reduced the translocation of NF‐κB to the cell nucleus during the subsequent exposure of chondrocytes to IL‐1β. This suggests that CN7:1h plays an anti‐inflammatory role by hindering the activation of NF‐κB, a key mediator in inflammatory responses. Given the general influence of small‐molecule drugs, the inhibitory effect of CN7:1h on NF‐κB aligns well with what is typically expected from compounds in this category (Figure 5). Similarly, CN7:1h displayed the hallmark features of small molecules by influencing mTOR activity, which triggered a cascade resulting in elevated autophagy levels. Note that this increase in autophagy is in line with the known effects of small molecules in modulating cellular behaviour and responses (Figure 4).

The OA‐related imbalance in molecular determinants was shown to decrease synthetic metabolism and increase degradative metabolism, leading to a decline in the quality of the ECM. It appears that this metabolic aberration sets off a cascade of events, including the large‐scale apoptosis of cartilage cells, cartilage degeneration and a corresponding decrease in joint function [33, 34]. Compounding this situation is the fact that chondrocyte apoptosis is often accompanied by a significant rise in MMPs and platelet‐reactive proteins, which further accelerates the degradative metabolism of cartilage cells and the degeneration of cartilage tissue [35, 36]. Our findings in the current study indicate that the small‐molecular drug CN7:1h plays a role in preserving the ECM, reducing the secretion of MMPs and the corresponding GAG loss. These effects are in line with other small molecules, which often allow for multi‐targeted interventions (Figure 1).

Ex vivo results revealed that IL‐1β insult led to a substantial loss of GAGs; however, pretreatment with 5 μM of CN7:1h for 2 h mitigated this loss (i.e. preserving GAG levels) (Figure 6). ACLT surgery in an animal model resulted in severe wear on the cartilage of surface at 3 months post‐surgery. Nonetheless, OARSI scores were significantly lower in the CN7:1h group than in the untreated ACLT injury group, thereby demonstrating the therapeutic efficacy of this compound (Figure 7). In ex vivo as well as in vivo settings, CN7:1h had promising effects on cartilage regeneration.

In our present study addressing limitations, we demonstrated how CN7:1h mitigated inflammation, apoptosis and extracellular matrix degradation in osteoarthritic chondrocytes. Our short‐term ex vivo models in human samples and in vivo models in rats all indicated the potential of CN7:1h as a treatment for OA. However, we lacked long‐term observations regarding its benefits for OA improvement. Additionally, our study did not encompass analysis of other derivatives of CN7:1h for further insights. Considering these limitations, it is essential to conduct further analyses of CN7:1h and its derivatives in relation to OA. Exploring their potential mechanisms and connection with CN7:1h or its derivatives warrants detailed ex vivo and in vivo experiments to thoroughly assess their applicability in clinical settings.

In conclusion, CN7:1h is a promising bioactive compound capable of harnessing the advantageous properties of small‐molecule drugs by modulating critical biochemical pathways, such as NF‐κB and mTOR, with beneficial effects on autophagy and apoptosis. Considering the broad range of biological activities and alignment with the characteristics of small‐molecule therapeutics, CN7:1h provides a viable new avenue for drug development aimed at treating degenerative diseases, including OA. Further studies will be required to elucidate its mechanisms of action and evaluate its suitability in clinical applications.

## Author Contributions

**Chih‐Chien Wang:** conceptualization (equal), data curation (equal), formal analysis (equal), funding acquisition (equal), investigation (equal), methodology (equal), project administration (equal), resources (equal), supervision (equal), validation (equal), writing – original draft (equal), writing – review and editing (equal). **Jeng‐Wei Lu:** conceptualization (equal), data curation (equal), formal analysis (equal), funding acquisition (equal), investigation (equal), validation (equal), writing – original draft (equal), writing – review and editing (equal). **Ya‐Wun Wu:** data curation (equal), funding acquisition (equal), investigation (equal), methodology (equal), validation (equal), writing – review and editing (equal). **You‐Hsiang Chu:** conceptualization (equal), funding acquisition (equal), investigation (equal), methodology (equal), resources (equal), visualization (equal). **Yi‐Jung Ho:** formal analysis (equal), investigation (equal), methodology (equal), resources (equal), validation (equal), writing – review and editing (equal). **Feng‐Cheng Liu:** formal analysis (equal), investigation (equal), methodology (equal), resources (equal), visualization (equal). **Yi‐Jen Peng:** conceptualization (equal), data curation (equal), formal analysis (equal), funding acquisition (equal), investigation (equal), methodology (equal), project administration (equal), resources (equal), software (equal), supervision (equal), validation (equal), visualization (equal), writing – original draft (equal), writing – review and editing (equal).

## Consent

All authors have agreed to publish in the Journal of Cellular and Molecular Medicine.

## Conflicts of Interest

The authors declare no conflicts of interest.

## Supporting information


Figure S1.

## Data Availability

The contents of the manuscript are shared upon request.
