# Supplementary material for: CN7:1h Alleviates Inflammation, Apoptosis and Extracellular Matrix Degradation in Osteoarthritis by Modulating the NF‐κB and mTOR Pathways
Source: J Cell Mol Med. 2025 Jan 28;29(3):e70368. doi: 10.1111/jcmm.70368 (PMC11774621; doi:10.1111/jcmm.70368)

**Supplementary Figure 1** Effect of CN7:1h on chondrocyte viability after incubation with 0, 1, 5, 10, 20, and 40 μM concentrations of CN7:1h for 24 or 48 hours. Data are presented as the mean ± SD from three independent experiments.


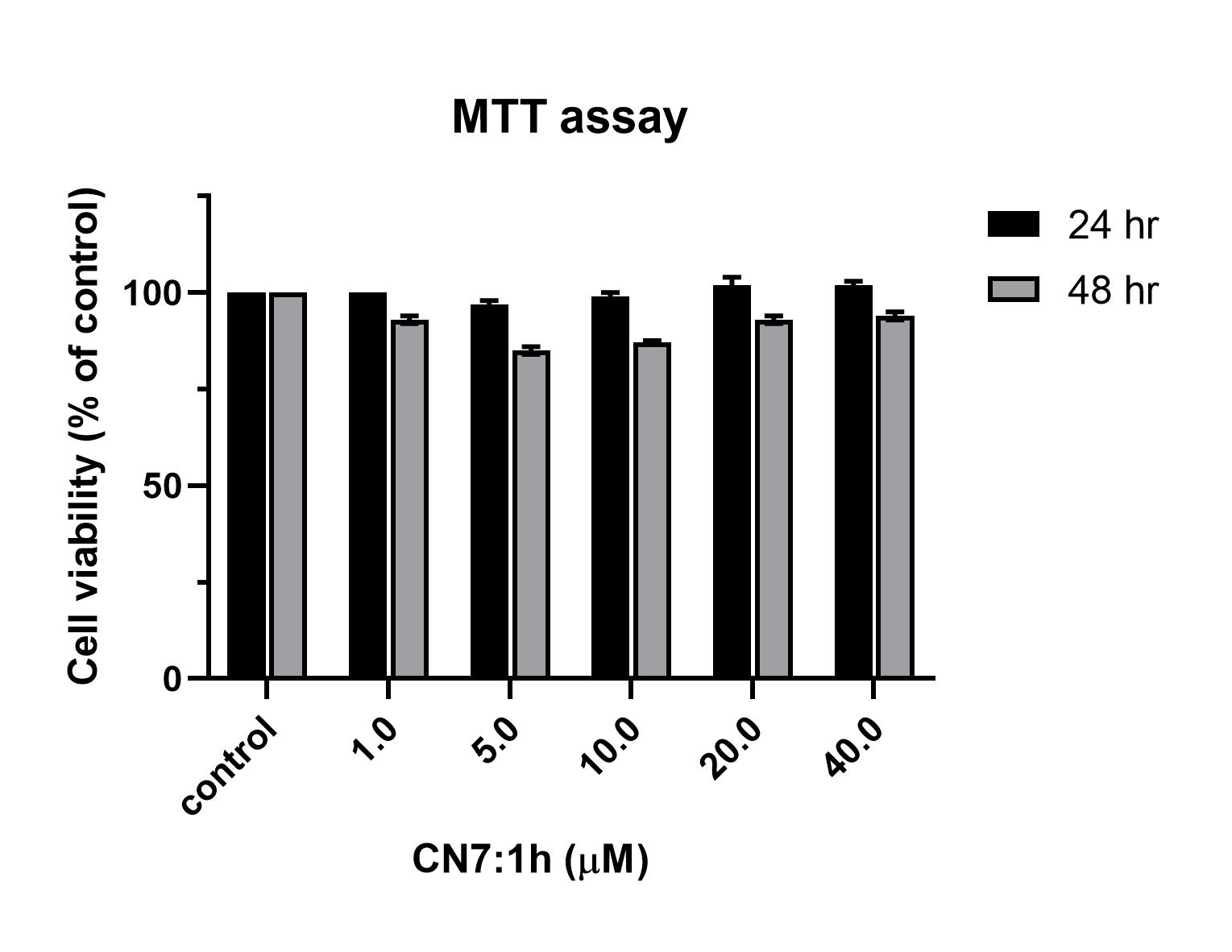

Supplement: Supplementary file 1 — Figure S1. [file JCMM-29-e70368-s001.zip › jcmm70368_sup-0001-FigureS1.docx]
