# Supplementary figures and images for: CN7:1h Alleviates Inflammation, Apoptosis and Extracellular Matrix Degradation in Osteoarthritis by Modulating the NF‐κB and mTOR Pathways
Source: J Cell Mol Med. 2025 Jan 28;29(3):e70368. doi: 10.1111/jcmm.70368 (PMC11774621; doi:10.1111/jcmm.70368)

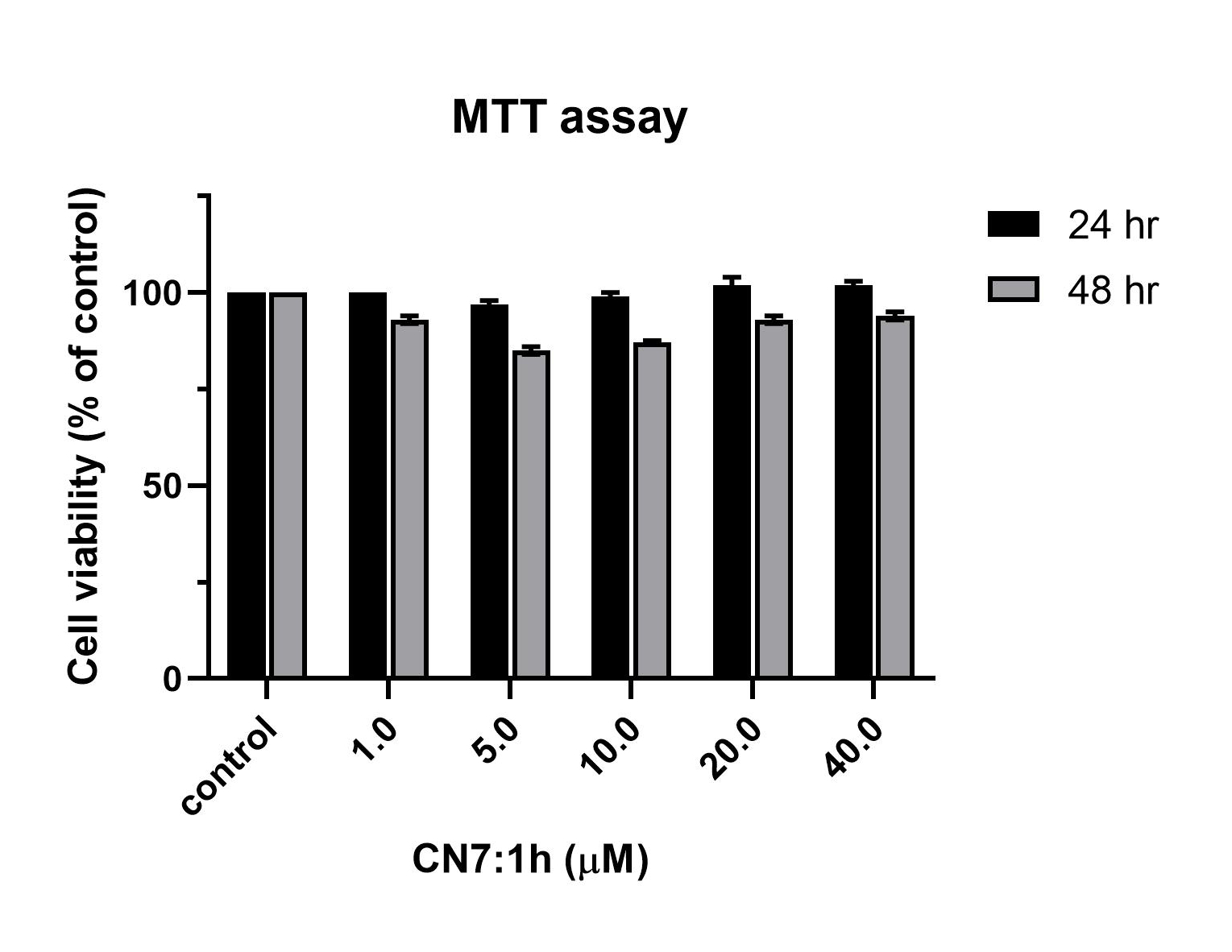

Supplement: Supplementary file 1 — Figure S1. [file JCMM-29-e70368-s001.zip › jcmm70368-sup-0002-FigureS1.jpeg]
